# First annulus formation in the European anchovy; a two-stage approach for robust validation

**DOI:** 10.1038/s41598-020-58174-5

**Published:** 2020-01-23

**Authors:** Gualtiero Basilone, Marco Barra, Rosalia Ferreri, Salvatore Mangano, Maurizio Pulizzi, Giovanni Giacalone, Ignazio Fontana, Salvatore Aronica, Antonella Gargano, Paola Rumolo, Simona Genovese, Angelo Bonanno

**Affiliations:** 1 Istituto per lo studio degli impatti Antropici e Sostenibilità in ambiente marino (IAS) - Consiglio Nazionale delle Ricerche (CNR), SS Capo Granitola, Campobello di Mazara, TP Italy; 2 Istituto di Scienze Marine (ISMAR) - Consiglio Nazionale delle Ricerche (CNR) SS di Napoli, Napoli, Italy

**Keywords:** Ichthyology, Marine biology

## Abstract

The age determination in fast-growing short-living species, such as European anchovy (*Engraulis encrasicolus*), has been widely recognized as a difficult task and bias introduced by readers leads to bias in reconstructing the population age structure. In this context, it is worth to note that age structure of fish population represents key information in fishery ecology and for stock assessment models. The uncertainty in estimating the age of the European anchovy by otolith reading is linked to the number of false-growth increments (checks) laid down before the annulus formation. While direct validation methods (e.g. mark-recapture, rearing, radiochemical dating) are difficult to implement specially for this short living species, the use of different indirect methods, supported by a coherent statistical approach, represents a robust and easier validation tool. A statistical modeling approach has been here adopted to assess the coherence of two well-known methods, namely Edge Analysis and Marginal Increment Analysis, in order to validate the first annulus formation in European anchovy. Both methodologies in two different yearly cycles converged toward the same result, thus confirming the annulus identification for the first year class. In addition, the completion dates of the checks and the first annulus were computed in order to gain a better insight into otolith growth dynamic. According to the species spawning period, the completion date of the first annulus falls in the summer period, while the first and second checks completion dates were mostly found in summer and winter respectively. General additive models using marginal increments as dependent variable showed a significant effect of the month, highlighting the presence of only one clear minimum in July/August, as well as specific relationships with condition factor and gonadosomatic index. Modeling the otolith edge morphology, the probability to find a hyaline band displayed in both years a similar shape, characterized by a minimum in July/August and higher values between November and January. The obtained results evidenced temporally coherent patterns providing a better insight in the otolith growth dynamic as well as a more robust validation of the first annulus formation in the European anchovy.

## Introduction

Otolith annual growth increments, composed of alternating opaque and translucent zones, are commonly used to determine the age of fishes since 1899^1^. However, counting annuli was sometime misleading in estimating the age of tropical or deep-sea adult fishes, mainly due to a more stable environment leading to more constant growth, thus resulting in less certain annulus formation^2^. Erroneous age estimates can negatively affect management of marine resources, since several biological variables such as growth-rate, mortality rate and productivity are obtained by the age structure of the population^3–5^. Age determination in short living species, such as anchovies, is further complicated because of the predominance of young fishes (0, 1 or 2 years old), which often show false rings or checks^6–9^. Correct age assignment mainly relies on a correct identification of year-classes. However, the year classes separation is difficult because anchovies have a long spawning season, which in the central Mediterranean Sea extends from April to October^10,11^. Although most fish age-estimation studies have assumed that growth increments occur annually, a substantial part of these did not test this assumption^12–14^. Even if not validated, these information are currently heavily used for anchovy stock assessment in Mediterranean and Atlantic waters^15,16^. There are various approaches for verifying age estimation methods^4,17^; edge analysis (EA) and marginal increment analysis (MIA) are among the most frequently methods employed^4,5^. These methods focus on incremental patterns of growth-band pairs throughout the year. They assume that, if growth bands are formed annually, the width and the density of the outermost increment will exhibit a yearly sinusoidal cycle when plotted against the month of capture. The EA is a qualitative assessment of the relative opacity and translucency of the edge of the otolith, whereas MIA typically compares the width of the last developing band to the width of a complete annulus. Both techniques are characterized by similar properties and, when used as validation method, provide valuables results, especially for fish species presenting annual periodicity in bands formation^4^.

Past studies have reported age validation based on MIA of young fish, but noted that the same ageing structure and/or approach provided incorrect ages mainly in older fish, suggesting that MIA is one of the few validation methods which is restricted to young, fast-growing fish^18–20^. Moreover, since proper age validation studies are still lacking for many species and study areas, age reading workgroups, specially addressed on European anchovy (*Engraulis encrasicolus*) age validation, recommended to seize upon any available method, which can corroborate the age interpretations as well as the dynamics of otolith formation (checks and true annual rings) by ages^13^. Although these techniques have sometimes been questioned^4^, their combined use, adopting a robust statistical framework and following rigorous criteria, may permit to evidence possible biases or inconsistencies between the different methods, and represents a useful tool for corroborating the correct annulus identification^4^. The use of combined information from different sources (fishery dependent or not) into a single holistic approach to corroborate or validate the annulus formation was already tested on different fish species, e.g. for Atlantic horse mackerel (*Trachurus trachurus*)^21^ and for red mullet (*Mullus barbatus*)^22^, as well as for both wild and reared anchovy in the waters of the Bay of Biscay^14,23,24^. The habitat conditions of the Bay of Biscay are characterized by higher riverine input nutrients and higher productivity^25^ compared to the oligotrophic Mediterranean waters^26,27^. Otherwise, the strong influence of habitat condition (e.g., primary production and temperature variability) in shaping otolith and growth rate was already detected for anchovy both in the Strait of Sicily^28,29^ and in the Bay of Biscay^24,30^. Therefore, validation studies for a single species should also consider the ecosystem variability, since it has been observed that the growth pattern could change among different habitat conditions^12,13^. In Mediterranean Sea, studies aiming at anchovy age validation are very scarce and pertain only to the NW area^31^. Indeed, the NW Mediterranean is among the most productive areas in the Mediterranean Sea^32,33^, thus not easily comparable with the oligotrophic nature of the study area (Strait of Sicily)^34,35^. Furthermore, none of the previous investigations used physiological information to support the results of the otolith analyses. Although two studies already validated the first annulus formation^14,24^, they were carried out through mesocosm experiments or by monitoring existing long time series of year-classes in catches. However both studies are not easy to be replicated in other areas (specially in Mediterranean Sea), since they rely on the availability of specific and complex infrastructures or long time series characterized by very strong year classes in successive yearly catches. In this study, we aimed to provide a different validation framework based on the combination of known methods coupled with robust statistical analysis, for areas/stocks where long time series of data or infrastructure facilities for rearing experiments are not available.

## Methods

### Sampling

Otoliths of European anchovy were collected from fishes of commercial catches in the Strait of Sicily from February 2015 to November 2016 at monthly intervals (Table 1). A random sample of a minimum of 50 fish per month was processed fresh in the CNR laboratory. For each fish, total length (TL, ±1 mm), total weight (TW, ±0.01 g), somatic weight (i.e. ovary-free weight, SW, ±0.01 g), and gonadic weight only for females (GW, ±0.01 g) were measured. Then, fishes were dissected in order to determine sex and reproductive phase, according to a six-phases scale^36^.

**Table 1:** Number of samples obtained by commercial landings in the Strait of Sicily during 2015 and 2016.

Sampling period		Total body length (cm)																	n° of fishes sampled
Year	month	9	9.5	10	10.5	11	11.5	12	12.5	13	13.5	14	14.5	15	15.5	16	16.5	17	
2015	February			1		5	5	5	5	5	5	5	5	2	2				45
	March		1	5	5	5	5	5	5	5	5	5	1	1					48
	April								1		8	9	6	4	7	6	2	1	44
	May						1	6	10	10	10	10	10	10	10	1	2		80
	June					2	6	10	10	10	10	10	10	2	1				71
	July						6	10	9	11	9	4							49
	August			2	9	10	10	6	7	10	10	10	10	1					85
	September					2	9	5	4	7	6	6	3						42
	October				3	4	10	10	10	9	8	9	10	4	1	1			79
	November								1	5	10	10	10	9	10	9	3		67
	December	1		10	10	10	10	10	10	5	4								70
2016	January	2	2	10	10	10	10	10	10	10	10	7	6			1			98
	February	1	2	3	10	10	10	10	10	10	9	7	3	3	1				89
	March				3	10	10	10	10	9	9	10	8	1					80
	April				7	14	20	20	20	12	13	10	9	9	1	1			136
	May			2	5	10	10	9	11	10	7	1	1						66
	June					4	10	10	10	10	10	2	1						57
	July				1	1	2	10	10	19	20	20	17	11	10				121
	August			2	2	9	10	10	10	9	3	1							56
	September				6	10	10	10	11	10	1								58
	October		2	2		1	9	13	21	28	30	30	23	15	12	6	1		193
	November			1		1	7	10	10	10	10	10	10	8	4				81
	*Total*	*4*	*7*	*38*	*71*	*118*	*170*	*189*	*205*	*214*	*207*	*176*	*143*	*80*	*59*	*25*	*8*	*1*	*1715*

No use of live animals has been required for this study and no specific permissions were needed for the sampling activities in all of the investigated areas because the species of interest is commercially harvested (neither endangered nor protected) and it was caught in areas where fishing is allowed.

According to ICES guidelines, otoliths (*sagittae*) were removed from a sub-sample of 5–10 individuals per size class at 0.5-cm length intervals^37^, cleaned, dried, and stored in black-plastic labeled moulds. The observations of entire otoliths were made under reflected light against a black background and using dissection microscopes with 25X magnification. Magnification has been increased (40X) near the otolith margin to improve the discrimination power of edge type morphology. For each specimen, both sagittal otoliths were laid in parallel with the sulcus facing down^12,13^.

### Age assignment

The method of age determination is based first on the interpretation of otoliths according to the prior biological knowledge of the annual growth pattern of the anchovy otoliths^13^. The basic information required for annual growth pattern identification and for age assignment are the date of capture and the conventional birthdate, which in the study area is set to 1^st^ July^13^.

The hyaline zones are usually formed in winter, but are not necessarily present from the beginning of the year^14,24^; therefore, the discrimination of true winter rings from checks is based on the knowledge of the typical annual growth pattern, seasonal growth of the edge (by ages), and of the most typical checks^13^. According to the typical annual growth of the otoliths, annulus width during the first, second and third year of life (corresponding to 0, 1, and 2 years old groups) decreases progressively.

**Figure 1:**
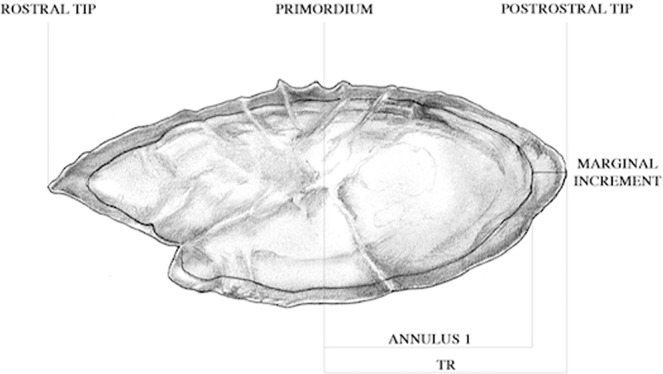
Schematic representation of the European anchovy whole otolith *sagitta*: TR = otolith total radius, and annulus 1 is the combination of one opaque plus one true hyaline zone (The authors acknowledge Barbara Bottini for drawing this image).

Therefore, according to the previous observations the adopted ageing criteria can be summarized as follows: each annulus consists of one opaque zone plus one hyaline ring; the age is equals to the number of true winter complete hyaline zones, corresponding to the expected annual growth pattern of the otoliths, excluding the marginal edge development of the year (Fig. 1).

### Otolith Edge state analysis (EA)

In order to assess the seasonal development of opaque and translucent zone within the population, the marginal growth of all the whole otoliths, collected from February 2015 to November 2016 (two complete cycles; 1715 otoliths), was examined also blinding the sampling date to the examiner.

**Figure 2:**
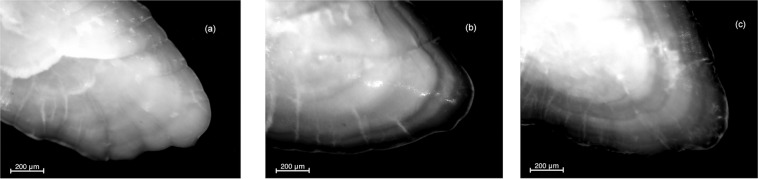
Morphology of the otolith edge for each type: (**a**) opaque margin (O); (**b**) hyaline margin (H); (**c**) semihyaline margin (OH).

The edge was classified as opaque (O), semihyaline (OH) or hyaline (H), according to the criteria described in literature^14^ and as shown in Fig. 2. Although the morphology of OH and H are quite similar, the criterion used to differentiate between them was the presence of the same edge around the whole otolith otherwise the OH is to be considered as opaque and thus merged with O^14^. According to this, the percentage of individuals showing different kind of edge were applied in the age 1group, for opaque (O), hyaline (H) and merged opaque and semihyaline (O + OH)/(O + OH + H) groups per month and sampling year.

### Translucent completion analysis (TCA)

In order to obtain information on the monthly distribution of the first annulus as well as on the translucent zones laid down before the first annulus (false ring or checks), the completion date of each translucent zone was reconstructed by backcalculating the fish length at the outer margin of each translucent zone, and then applying the von Bertalanffy growth function with L_inf_, k and t_0_ parameters specific for the study area^28^.

The translucent zones were labeled sequentially from T1 to T3 where the latter represents the zone identified as first annulus by readers, while T1 and T2 are those rings laid down in the inner zone of the otolith, which are generally one or two clear zones. In particular, starting from length at capture (Lc) and radius measurement, the length of fishes at complete formation (i.e. the outer margin) of each translucent zone (L*i*) were obtained by backcalculation^38^:

$$Li=\frac{(a+bTi)}{(a+bTR)}Lc$$

where a and b are the parameters of the linear relationship between otolith radius (TR) and fish length at capture (TL), while Ti represents the radius from the core to the outer margin of *i-*th translucent zone.

Starting from backcalculated Li for each translucent zone, the corresponding ages at completion were then obtained by using the growth model estimated for the whole anchovy population in the study area^28^.The use of the latter published model is justified by the narrow size range of the present dataset, preventing a robust estimation of L_inf_, t_0_ and k parameters. Finally, the completion date in each fish (F*i*) was computed according to the following formula:

$${Fi}=capture\_date-(Ac-Ai)$$

where A*c* is the age at capture (in days) and A*i* is the age (in days) at *i*-th translucent zone completion.

### The otolith marginal increment analysis (MIA)

Individual data on the seasonal development of the marginal zone were obtained according to the procedure previously described for the translucent analysis. Despite digital measurement is often used in MI studies, in the present study it was preferred to take the radius measurements, just after the annulus zone has been identified on the whole otolith, by using a micrometer fitted in the eyepiece^39^. This procedure may ensure to take the measurement of the checks or true annuli just after the identification by readers, therefore potentially reducing sources of bias (e.g. misidentification of the annual zone on digital system). According to the adopted age reading protocol^12,13^, the whole otolith under a dissecting microscope was used to assign properly the true annual ring. The total radius length (TR) of the whole otolith was measured along the longest axis from the core to the post-rostral outer edge of the otolith. The intermediate radii (R*i*) at each complete translucent zone corresponding to annulus formation were also measured (Fig. 1). The MIA technique^3,40^ was applied by calculating the total radius (TR) minus the radius of the last complete hyaline zone (R*i*) in a selection of one-year-old fishes and MI was expressed as a proportion of the measurement of the previous last complete annulus, i.e. marginal increment ratio (MIR) (Fig. 1).

In order to give more robustness to MIA, four prerequisite were satisfied^4^: (1) blinded sampling date to the examiner; (2) a minimum of two complete cycles (years) have been examined, in accordance with accepted methods for detecting cycles; (3) the results were objectively interpreted by means of a statistical test, which may show significant differences among some or all of the seasonal groups in each of the cycles examined; (4) MIA has been restricted to age 1 group, avoiding age 0 and older ages (age 2 and age 3). Therefore, the validation results should be considered to be age-specific.

### Reproductive and body condition cycle

In order to evaluate the temporal link among the reproductive cycle, somatic conditions otolith edge and microincrement formation monthly patterns, gonadosomatic (GSI) and a condition factor (CF) were computed per age group. Although some authors questioned about the usefulness of the gonadosomatic index as a proxy of reproductive potential, because it can be influenced by fish length^41–43^, several studies validated its applicability for such investigation^44,45^, highlighting also its validity in batch spawning species^10,46^. In the present work, GSI was calculated according to the equation described by Bougis^47^:

$$GSI=\frac{GW}{SW}\ast 100;$$

In order to investigate the otolith growth pattern formation, with respect to the annual cycle of fish condition, the Fulton’s equation was used for condition factor:

$$CF=\frac{SW}{T{L}^{3}}$$

In both equations, the SW was preferred to the TW to reduce the effect of GW seasonal variability^10^.

### Statistical analysis

Generalized Additive Models (GAM)^48^ were used in order to evaluate the presence of significant effects on otolith structure of month, GSI and CF. GAMs represent a generalized form of linear models that allow dealing with complex relationships. Due to the differences in the number of observations between the two years, models were fitted separately by years. In particular, the probability to find a hyaline band, as well as MIR values, were modeled as function of month, GSI and CF.

In modeling the probability to find a hyaline band, the categorical variable related to the classification of otoliths bands (H, O, OH) was transformed in a binary variable by assigning 1 to the H bands and 0 in all other cases (O and OH) and a binomial error distribution family with logit link was used. On the contrary, a Gaussian family with identity link was adopted to model the MIA values. The best model was selected according to Wood’s guidelines^49^ and adopting a backward strategy. All final models were checked for residuals autocorrelation and concurvity.

All statistical analyses were carried out in R statistical environment^50^. GAMs were fitted by using “mgcv” package^51^.

## Results

### Age structure

**Figure 3:**
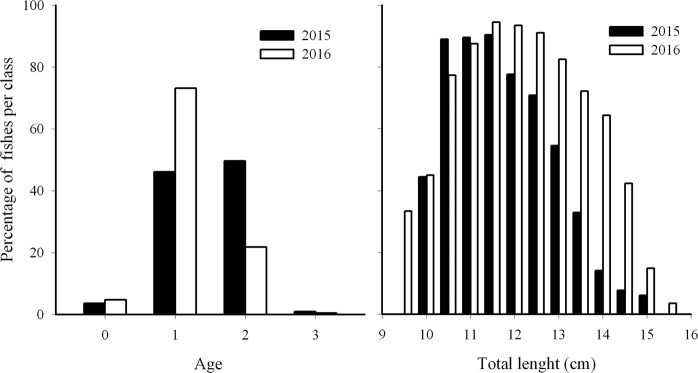
Age structure of European anchovy for each analyzed year from the GSA 16 stock: (**a**) proportion for age class; (**b**) proportion of age 1 fishes for length class in 2015 (n° 313) and 2016 (n° 757).

In the two years considered in the present study, otolith readings and age assignment of the anchovy stock displayed a quite different age structure where the age classes 1 and 2 accounted for most part of the population (Fig. 3a). Most likely, the age 0 is not fully representative of the recruitment, because size classes of age 0 fishes were mostly below the minimum legal size and the samples came from commercial landings. Despite the dissimilarities recorded in the age structure by year, the length frequency distribution between the two analysed years (Fig. 3b) is not significantly different (Kolmogorov Smirnov test p > 0.1).

### Reproductive cycle and somatic condition

**Figure 4:**
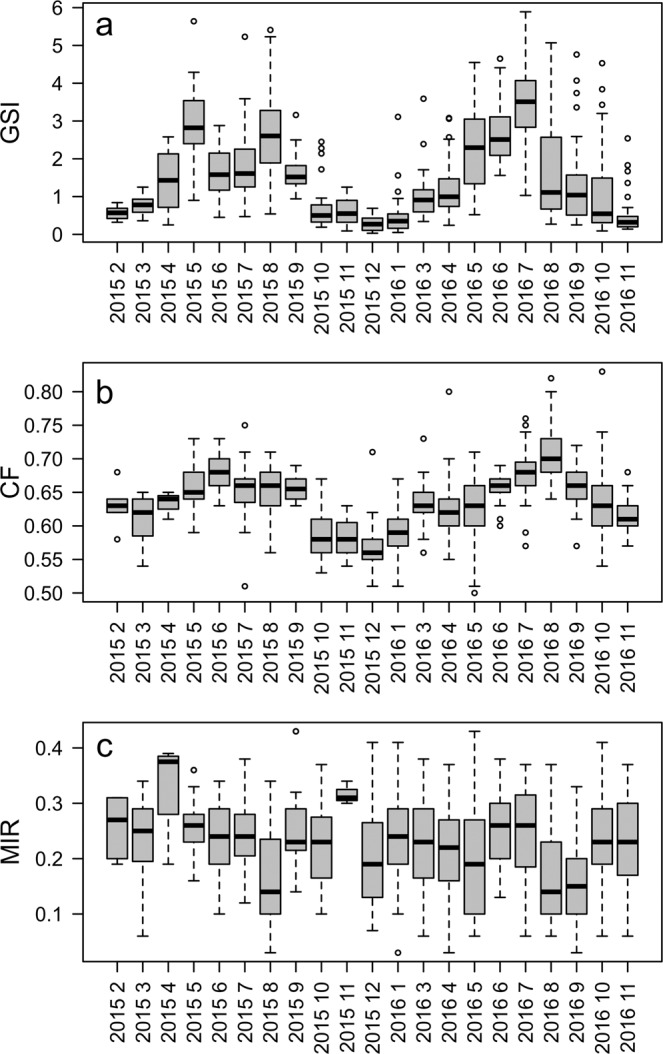
Boxplot of temporal trend (monthly) for fishes of age class 1: (**a**) gonadosomatic index (GSI); (**b**) condition factor (CF); (**c**) and otolith marginal increments (MI).

The monthly boxplot of GSI from age 1 class showed that the reproductive season extends mainly from May to July-August, and started decreasing along autumn and winter (Fig. 4a). The GSI values suggested an earlier seasonal resume of the reproductive investment, since GSI values at the start of reproductive season in May were already similar to those estimated in August; while in 2016 the highest value was recorded in July. The visual inspection of the CF monthly pattern obtained for the age 1 class showed a trend similar to GSI with lower values between October and March, and maxima between June and August (Fig. 4b).

### Otoliths edge seasonal growth pattern (EA)

**Figure 5:**
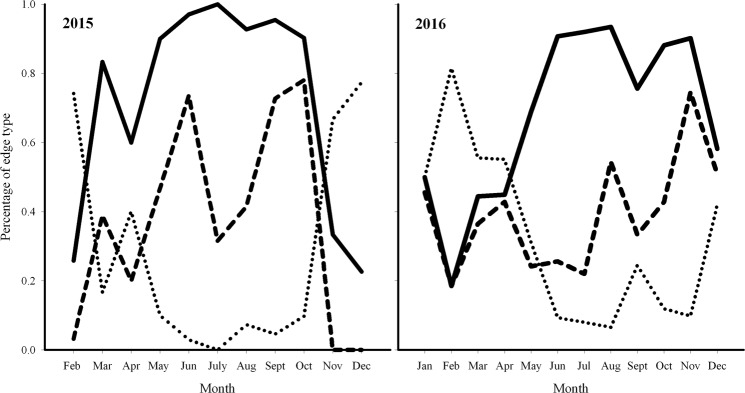
Monthly evolution (%) of edge morphology in 2015 (left panel) and 2016 (right panel) for fishes of age class 1: opaque plus semihyaline edges (O + OH, merged according literature^14^, solid line); hyaline edge (H) (dotted line); opaque edge (O) (dashed line).

**Figure 6:**
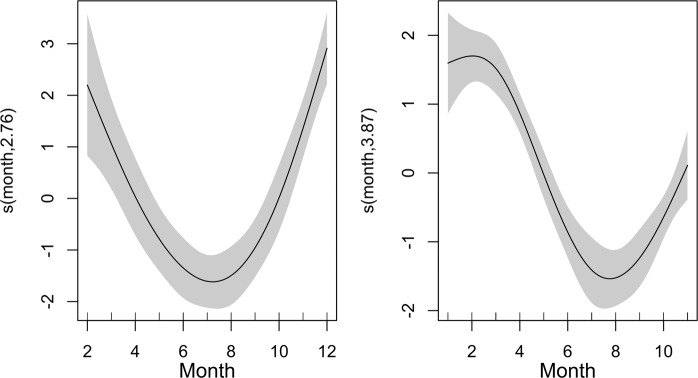
Plots of the fit (GAM) between the probability to find a hyaline margin and month. Shaded (light-grey) regions represent the confidence bands (±2 SE) for smooths. Model-predicted presence of translucent otolith edges in 2015 (left panel) and 2016 (right panel).

The inspection of the morphology of the otolith edge in 1 year old fishes showed a clear yearly cycle (Fig. 5). In both years, the opaque growth (O + OH) resumes in March, and until September-October in above 90% individuals. The application of GAM to model the probability to find a hyaline band using CF, GSI and month as predictor evidenced a significant effect (p < 0.001) for month only in both years. Explained deviance was 43.6% in 2015 and 18.6% in 2016. Model residuals were checked for autocorrelation and analysis results evidenced the presence of a weak autocorrelation for specific lags. Anyway, the maximum observed significant correlation values were respectively 0.25 in 2015 and 0.23 in 2016 model and were thus considered as negligible. Opposite (but coherently) to the opaque growth pattern, the shape of the relation of hyaline margin occurrence showed in both years a common temporal pattern (Fig. 6), with a clear minimum in July/August, and higher values between November and January.

### Translucent zone completion analysis (TCA)

The back-calculation estimated parameters, used to obtain the fish length at hyaline complete formation, were computed by fitting a linear model for each year (in order to avoid bias due to the larger number of fishes in 2016 dataset). The obtained relationships were TL = 69.66*TR + 20.94 (r^2^ = 0.74) in 2015 and TL = 71.22*TR + 19.99 (r^2^ = 0.67) in 2016.

**Figure 7:**
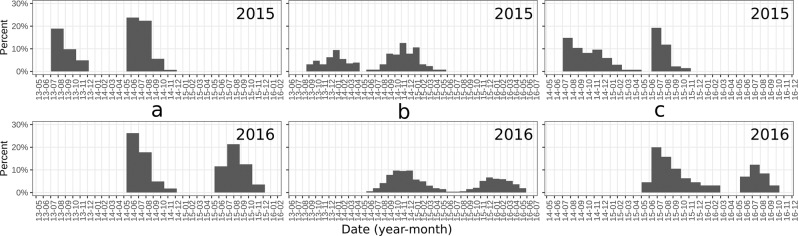
Completion date frequency distribution (TCA) of the analyzed translucent zones showing two cohorts per sampling year respectively in 2015 top panel, and 2016 bottom panel. The three different translucent zones were plotted respectively T1 in panel (a); T2 in panel (b); and T3 in panel (c).

The completion date frequency distribution of the three translucent zones (T1, T2 and T3) identified two different cohorts in both 2015 and 2016, which present quite similar monthly distribution in the two years. Namely, the ring closest to otolith core (T1) spanned from May-June to October-November with clear peaks in June, July and August (Fig. 7a). The second translucent zone (T2) completion spanned mainly between September and April of the subsequent year, with maximum in December-January (Fig. 7b). Finally, the third translucent zone (T3) distribution showed starts to be completed not before May, while ending in October until February of the following year. Higher proportions of T3 completion was recorded in July-August (Fig. 7c).

### Marginal increment width seasonal variability (MIA)

Marginal increment analysis of otolith measurements from the two analyzed years was obtained only for age 1 class anchovy, according to the recommendations to obtain a robust application of MIR to validate annuli^3,4,52^. Analysis of the MI variability through the year revealed that the minimum of marginal width, which is associated to the beginning of the new annulus formation, was recorded in August in both years (2015 and 2016), even if in September 2016 the minimum of MI values still persist (Figs. 4c and 6).

**Figure 8:**
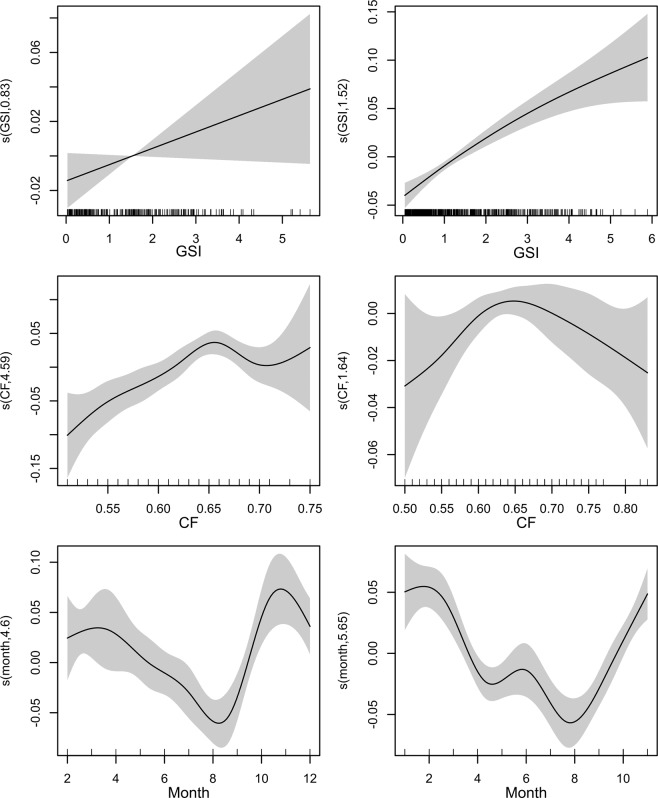
Plots of fitted GAMs in 2015 (left panels) and 2016 (right panels) of the marginal increment ratio (MIR) considered as dependent variable and the three considered factors: gonadosomatic index (GSI), condition factor (CF), and month. Shaded (light-grey) regions represent the confidence bands (±2 SE) for smooths.

The application of GAM confirmed a significant (p < 0.05) effect of month, GSI and CF in both years. Indeed, the best model was in both years the one considering all the terms, while all the reduced models (i.e. the ones considering a lower number of explicative terms) were characterized by a strong decrease in explained deviance. Both final models were checked for residuals autocorrelation and concurvity. Significant maximum absolute autocorrelation values for residuals were 0.17 in 2015 and 0.1 in 2016 and were considered as negligible. Estimated concurvity ranged between 0.24 and 0.28 for the 2015 model and 0.2 and 0.25 for 2016. The deviance explained by the models was 19.3% in 2015 and 16.7% in 2016, highlighting a certain amount of intrinsic variability mainly due to the large extension of the anchovy spawning period (about 6 months). The shape of relationships was quite consistent between the two years (Fig. 8), clearly confirming the presence of a well-defined minimum of the MI values in august. CF also showed a consistent pattern with respect to the MIR, highlighting a positive relationship up to CF value between 0.60 and 0.70 where a plateau was reached, while an almost linear positive relationship was observed for GSI.

## Discussion

In this study, EA and MIA were used to validate the periodicity of otolith increment formation in European anchovy. Annulus formation for this species in the Strait of Sicily occurred in August for both years, even if in 2016 insisted one month more, probably due to a shift in the spawning period in 2016 compared to 2015, as indicated by the GSI monthly evolution (Fig. 4a) and by the slightly different position of the minimum in GAMs (Fig. 6). Since minimal marginal increments on otoliths occurred only once per year (Fig. 8), the annulus was accepted as valid indicator of age. Moreover, the change in relative frequency of each edge zone (EA) plotted against months, as with MIA, confirmed that the frequency was one cycle per year (Fig. 5). The time completion of the first translucent zone showed that in most part of samples T1 is already laid down around summer, that corresponds to the peak of spawning period for this species in the study area^10^. This first translucent zone could be associated to the metamorphosis, which generally occurs between 40 and 60 days after hatch^29^. This evidence is in agreement with the time elapsed from the beginning of the spawning period (April) in the study area^10^.

The TCA for T2 suggested that its formation would start in spring-summer, since it appears to be completed mostly from September-October (Fig. 7b). Consequently, this should not to be considered a true winter ring (annulus).

The completion distribution of the T3 showed a time shift compared to Bay of Biscay anchovy^24^, where the hyaline growth in anchovy juveniles was observed ending in April. Anyway, it is worth noting that the spawning peak of anchovy in the Bay of Biscay occurs between May and June^53^; thus the first annulus formation is completed (T3) one month before the spawning peak. In our study area a similar pattern was observed, since the first annulus start to be completed in May/July, with the spawning peak occurring in late July/August. Numerous studies have reported large variations in seasonality of otolith band formation between species^54^, as well as between populations within the same species^55,56^, mainly linked to somatic growth^57^, reproduction^58^, photoperiod^57^, and temperature^54,59,60^. In this context, the observed time shift in the completion date of the first winter ring with respect to the one observed in Bay of Biscay anchovy could be due to the combined effect of temperature characterized by higher minima (SST estimates in the study area was never lower than 14 °C^10^, also during the study period; data not shown) and the shift in the spawning season. Such temporal lag is also indicated by EA, showing the resume of opaque growth zone to begin in March and above 90% of specimens with opaque margin in July (Fig. 5). Accordingly, the MIA indicated that the annulus formation resulted mostly complete in August (Fig. 8c), as also suggested by the hyaline edge monthly pattern (Fig. 6).

A minimal marginal increment on otoliths occurring only once per year has been observed in literature also for anchovy of the Chesapeake Bay^61^. Although different from the European anchovy, such species in the Chesapeake Bay presents its birthdate on mid July and the annulus formation has been established one month apart (May-June) of the following year. Other studies dealing with MIA on other pelagic species (*Sardinops sagax* and *Trachurus trachurus*), conducted in South African waters, showed the use of marginal increment technique as useful tool for annulus validation^62,63^. A proper validation study would necessitate several evidences from different methodologies and, if possible, by a direct approach (e.g. mark-recapture, modal progression analysis of strong year-classes in the catches, etc.). For European anchovy, Aldanondo *et al*.^24^ focused their study on validation by means of daily ring analysis in juveniles. Another important result for this species was obtained by Uriarte *et al*.^14^ that adopted a specific approach, based on the opportunity to detect a particular abundant year class along the time series of landings data, which enabled the authors to follow this cohort for the life span of the species. The validation framework, proposed in the present study, based on the combined use of MIA and EA, and supported by statistical modeling and translucent zones completion frequency distribution, permits to obtain a robust corroboration of first annulus formation, even when data like those proposed by previous studies are not available (i.e. daily rings and/or long time series of landings data with a particularly abundant cohort).

Although previous studies exist for European anchovy, such investigations are only carried out on fishes from the Bay of Biscay^14,23,24^ and from the NW Mediterranean area^31^. In both these areas the habitat conditions are characterized by higher riverine input nutrients and higher productivity^25,31^ than the oligotrophic waters of the study area and of the most part of the Mediterranean Sea^26^. The Strait of Sicily is considered an oligotrophic area^34,35,64^, where the enrichment of the upper water layers is associated with upwelling phenomena, which allow nutrient inputs from deeper waters^27,65^. Literature widely reported the variability in somatic growth linked to habitat variability e.g. ^24,28,29,64,66,67^. The growth rate of the anchovy in the Strait of Sicily was at the low end of the range observed for this species among different areas, also including the Bay of Biscay and NW Mediterranean^24,31^. Taking into account such aspects, validation studies should be carried out not only at species level but also on different populations of the same species inhabiting areas with different environmental conditions.

Each age validation method has advantages and disadvantages which would be expected to affect the results. In the present study, the analyses were carried out according to the recommendations to obtain sufficient rigor for the validation of the first annulus formation cycle^4^. Both methodologies (EA and MIA) in the two different years converged toward the same result, thus confirming the annulus identification to be correct at least for the first annulus formation. The convergence of independent methods toward the same result, supported by a statistical approach, represents in this context a good indicator to determine the robustness of the age validation. The relative scarcity of validation studies on wild fish therefore suggests that all available data and methods should be used to provide valuable support for age validation in the meantime that new validation studies are designed and implemented to confirm the accuracy of an age estimate^4,13,14^.
